# What Really Causes Necrotising Enterocolitis?

**DOI:** 10.5402/2012/628317

**Published:** 2012-12-17

**Authors:** Thomas Peter Fox, Charles Godavitarne

**Affiliations:** General Medicine, King's College Hospital, London SE5 9RS, UK

## Abstract

*Background*. One of the most serious gastrointestinal disorders occurring in neonates is necrotising enterocolitis (NEC). It is recognised as the most common intra-abdominal emergency and is the leading cause of short bowel syndrome. With extremely high mortality and morbidity, this enigmatic disease remains a challenge for neonatologists around the world as its definite aetiology has yet to be determined. As current medical knowledge stands, there is no single well-defined cause of NEC. Instead, there are nearly 20 risk factors that are proposed to increase the likelihood of developing NEC. *Aims and Objectives*. The aim of this project was to conduct a comprehensive literature review around the 20 or so well-documented and less well-documented risk factors for necrotising enterocolitis. *Materials and Methods*. Searches of the Medline, Embase, and Science direct databases were conducted using the words “necrotising enterocolitis + the risk factor in question” for example, “necrotising enterocolitis + dehydration.” Search results were ordered by relevance with bias given to more recent publications. *Conclusion*. This literature review has demonstrated the complexity of necrotising enterocolitis and emphasised the likely multifactorial aetiology. Further research is needed to investigate the extent to which each risk factor is implicated in necrotising enterocolitis.

## 1. Background

One of the most serious disorders and the single most serious gastrointestinal disorder occurring in neonates is necrotising enterocolitis [1–3]. This enigmatic disease remains a challenge for neonatologists around the world as its definite aetiology has yet to be determined. It is recognised as the most common intra-abdominal emergency effecting neonates and it is the leading cause of short bowel syndrome [4, 5]. Necrotising enterocolitis is characterised by the bowel wall necrosis of variable thickness, which leads to perforation in up to one-third of cases [6].

The condition was first described by Paltauf in 1888 but the term “necrotising enterocolitis” was used for the first time by Schmid and Quaiser in 1953 [7, 8]. Since then, there has been a tremendous increase in the incidence of NEC. This has been attributed largely to two factors. The first is the increase in the number of premature births by Caesarean section for therapeutic reasons resulting in the delivery of premature babies. The second reason is due to the advancement in technology of neonatal care, for example, intensive care units and surfactant therapy have enabled most premature babies to overcome a number of previously fatal conditions and survive, making them highly susceptible to developing NEC [9, 10].

## 2. Epidemiology

The incidence of NEC varies not only from country to country but also among different NICUs in the same country. Research by the National Institute of Child Health and Human Development observed variations from 4 to 20% in the prevalence of NEC at centres across northern America, suggesting that iatrogenic factors may play a large role. These have yet to be identified [11, 12]. The incidence in the population as a whole is estimated to be between one to three cases per 1000 live births. However, NEC occurs in 2–5% of very low birth weight infants (VLBW) and in 1–8% of neonatal intensive care unit admissions (NICU) [13]. A survey carried out in 1994 by the British Paediatric Surveillance Unit reported 300 new cases of NEC in the UK in one year with an overall death rate of 22% [14]. Median gestation was 29 weeks and 65% of babies weighed under 1500 g. However, 12% of babies that developed NEC were born at term [15–17].

## 3. Pathology 

NEC can arise in any area of the GI tract; however, the most common sites are the terminal ileum, caecum, and ascending colon [18]. Pneumatosis intestinalis (the presence of submucosal and subserosal gas within the bowel wall) is the most characteristic appearance of the gut radiologically and on laparotomy. The gas is mainly nitrogen and hydrogen formed by gas producing bacteria in the GI tract [19]. Histologically, the earliest signs of NEC are necrosis of the mucosa with microthrombus formation, leading to oedema, patchy ulceration and haemorrhage [20].

Important elements in modulating the damage that results from NEC are the inflammatory cytokines interleukin 1, 3, 6, tumour necrosis factor (TNF), and platelet activating factor (PAF). Indeed, these markers can be used to predict the severity of the disease [21]. PAF is a proinflammatory cytokine and has been shown to be of a particular importance [22]. Stool levels of PAF rise sharply with the onset of NEC and the administration of PAF to rats in a hypoxic atmosphere has shown to induce NEC [23]. Furthermore, PAF antagonists reduce the incidence and severity of NEC in rats [24]. This may also explain why enteral feeding is a risk factor for NEC; as stool concentrations of PAF increase, enteral feeding is commenced [25].

## 4. Aetiology

Many potential risk factors have been explored in relation to the aetiology of NEC; however, the definite aetiology still eludes modern medical research. It is possible that no individual factor is sufficient to precipitate NEC. Risk factors identified so far are shown in Table 1, [26].

## 5. Literature Review

At present there is no single, well-defined cause of necrotising enterocolitis. Instead, we have nearly 20 risk factors that are proposed to increase the likelihood of developing NEC [26]. In this literature review, the various risk factors that are thought to be most significant will be discussed as well as certain parameters that may change with the onset of NEC.

## 6. Birth Weight and Prematurity

Low birth weight and prematurity have been identified as among the most important risk factors for NEC [27–30]. NEC occurs in up to 5% of VLBW infants and the median gestation is around 29 weeks [31]. 65% of cases had a birth weight under 1500 g and only 12% of cases are born at term. Indeed, preterm infants born under 30 weeks who develop NEC usually have no other risk factors [32].

## 7. Gender

Carter and Holditch-Davis published a study in 2008 involving 134 preterm infants which concluded that there was no relationship between gender and NEC [33]. Ballot et al. conducted a study of 474 preterm infants in Johannesburg and were able to determine minor discrepancies between gender incidence of NEC (OR 3.21; 95% CI 1.6–6.3) [34]. However, most research has concluded that no differences are noted in the incidence of NEC according to gender [35]. Therefore gender is not thought to be a risk factor for NEC.

## 8. Neonatal Hypoxia

Recurrent apnoea, respiratory distress, assisted ventilation, and umbilical artery catheterisation are all known to contribute to hypoxic events in the first few weeks of life [26]. Palmer and Thomas conducted a study in 1987 which identified all of the above as risk factors for VLBW infants [36]. An adverse intrauterine environment can lead to chronic foetal hypoxia and IUGR. This can result in a diversion of cardiac output away from the gut which could precipitate necrotising enterocolitis [37].

In the term infant with NEC, risk factors for gut hypoxia are invariably present. NEC may follow severe generalised hypoxia, maternal cocaine use, or exchange transfusion [38]. An early study by Goldberg and Thomas into 5 infants born at term who developed NEC in 5–7 days found that they had all suffered from severe hypoxia during birth [39].

The use of hyperbaric oxygen therapy trialled in rat models has been shown to significantly reduce the severity of NEC [40]. However, further research is required before this can progress to human trials.

## 9. Maternal Milk versus Formula Milk

Breast milk protects against NEC [41]. In a study by Lucas and Cole involving 926 preterm infants, the exclusively formula fed group had a 6–10-fold increase in the rates of NEC than those fed with breast milk alone and 3 times the rates of those fed on a mixture of breast milk and formula. In the same study, delayed enteral feeding was also associated with a decreased incidence of NEC [42]. A systematic review conducted in 2003 also found a 3-fold increase in the likelihood of developing NEC if the infant is solely fed on formula [43]. When the general health of the infant is considered, breast milk also appears to be far superior [26]. There are significant benefits to infant host defence; sensory-neural development, gastrointestinal maturation, and some aspects of nutritional status are observed when premature infants are fed with their mothers' own milk. A reduction in infection-related morbidity in human milk-fed premature infants has been reported in nearly a dozen descriptive, as well as some RCTs in the past 25 years [44].

Trophic feeding is defined as the use of minimal enteral feeds (continuous drip of less than 1 mL/hour) with parenteral nutrition. This has been shown to lower the incidence of feeding intolerance, shorten the duration of time to regain birth weight, and decrease the incidence of NEC [45, 46]. Little is gained nutritionally from trophic feeding; however, some degree of nutrient exposure is essential even to the immature gut to prevent intestinal mucosal atrophy [47]. A recent trial by Berseth and Bisquera investigating the benefits of trophic feeding over standard milk advancement had to be stopped because babies receiving the standard regimen had a higher rate of NEC (10% versus 1.4%) [48]. Henderson et al. suggested that the duration of trophic feeding and the rate of the advancement of feed volumes are key modifiable risk factors for NEC in preterm infants [49].

Manipulating the chemical composition of formula milk, by reducing protein content, adding prebiotics, growth factors, or secretory IgA, can modulate an intestinal development. This has been suggested to reduce the differential responses between breast-fed and formula-fed neonates [50].

Mothers of preterm infants produce milk that has a higher protein content, higher caloric density, and higher calcium and sodium content than milk from mothers who deliver at term [51]. This matches (to a certain extent) the increased needs of the premature infant. This has led to the development of specialised preterm infant formulae. 

When infants on the NICU do not show adequate growth and weight gain there are a number of things that can be considered: firstly, increasing the volume of feeds. When the volume cannot be advanced any further there are two options: use donor hind milk or add commercial fortifiers. There have been a number of reports of infants developing NEC following the addition of commercial fortifiers [52, 53].

A key component of feeds that may be a modifiable risk factor for NEC is osmolarity [54]. There is a concern that additives to maternal milk may alter the osmolarity and hence remove the protective effect against NEC [55]. A number of studies have found that human milk and formula milk interact to induce a rapid increase in osmolarity higher than that which would be expected from composition alone. This rise could be explained by the amylase activity of human milk, inducing the hydrolysis of the dextrin content of formula milk, leading to small osmotically active molecules of oligosaccharides. Routine additives can significantly increase the osmolarity of EBM to levels that exceed current guidelines for premature infant feeding. The high osmolarity of fortified human milk should therefore be considered in the nutritional management of preterm infants [56, 57].

## 10. Blood Results

Anaemia is associated with an increased risk of developing NEC [26]. Blau et al. also found that neonatal transfusion of packed red blood cells could be a trigger for NEC [58]. A further study in December 2010 by Josephson et al. concluded that PRBC transfusions were merely a marker of disease severity and that there was no correlation with NEC [59]. Such conflicting views, published within weeks, of each other highlight the need for a further research in this area.

Polycythaemia was first suggested as a risk factor for NEC in 1975 [60] although initial studies failed to confirm this suggestion [61]. A review of 36 premature infants born over a period of 5 years dismissed polycythaemia as a risk factor in the development of NEC [62]. However more recently, many studies have identified increased incidences of polycythaemia in groups of infants that develop NEC compared to a control group [63–66]. The aetiology of neonatal polycythaemia is related either to intrauterine hypoxia, or secondary to fetal transfusion. There is a linear relationship between hematocrit and viscosity until 65% after which it is exponential. It is the increased viscosity of blood that is responsible for reduced mesenteric perfusion [67, 68]. As viscosity increases so does the tendency to form microthrombi which can further impede mesenteric perfusion. At present, polycythaemia is widely regarded as a significant risk factor and current guidelines recommend the prompt diagnosis and management to avoid adverse outcomes [69].

Platelets are an acute phase reactant, and thrombocytosis can represent physiologic stress to an infant. However, acute NEC is more commonly associated with thrombocytopenia (<100,000/*μ*L) [70]. The evidence behind thrombocytosis directly causing NEC is scanty; however, it stands to reason that a thrombogenic state could reduce mesenteric blood flow [71].

80–90% of cases of NEC are associated with thrombocytopenia to some degree [72]. Kenton et al. conducted a study on 91 infants and found that severe thrombocytopenia is a valuable prognostic indicator of mortality associated with NEC and may influence management options. Thrombocytopenia may become more profound in severe cases that become complicated with consumption coagulopathy [73]. This report suggested that prospective studies of infants with early and severe thrombocytopenia may help determine the optimal timing of laparotomy in infants with NEC. Ververidis et al. concluded that a platelet count of less than 100 × 10^9^/L or a sudden fall in platelets was a poor prognostic indicator [74]. It therefore seems that while thrombocytosis is a risk factor because it induces a thrombogenic state that may impede mesenteric blood flow, thrombocytopenia is perhaps more useful as a prognostic indicator.

## 11. Dehydration/Electrolyte Disturbances 

Dehydration is a risk factor for NEC [75]. When severe, it increases the viscosity of blood. Increased viscosity has been shown to decrease mesenteric perfusion and hence may precipitate NEC. There are numerous case studies demonstrating this phenomenon [76, 77]. Interestingly, hyperhydration has also been shown to increase the risk of NEC in a Cochrane review [78]. The conclusions to this review were that the careful restriction of water intake is required so that physiological needs are met without allowing significant dehydration.

## 12. Acute Phase Proteins

C-Reactive Protein (CRP) is one of the acute phase reactants which has been proven to be a useful marker of inflammation, not only in the gastrointestinal system but also systemically [79]. CRP is usually elevated in NEC [26]. It has not been found useful in predicting the onset of NEC as the rise in CRP appears to lag behind the clinical onset [80]. This study showed that CRP has increasing sensitivity but remains a nonspecific marker of NEC [81]. Many studies have analysed the serial changes in CRP before and after the diagnosis of NEC and it is thought that it may be more useful in predicting outcome and determining severity [82]. A retrospective analysis of data ranging from January 2001 to July 2006 on preterm (gestation < 32 weeks) neonates with definite NEC found that serial changes in CRP may predict the progression to surgery as well as death [83].

There are many other neonatal conditions which are associated with an elevated CRP: septicaemia, meningitis, urinary tract infection, pneumonia, meconium aspiration syndrome, or presumptive infection [84, 85]. 

The usefulness of CRP in diagnosing NEC seems to be in conjunction with radiographic changes, classically pneumatosis intestinalis. CRP is also useful in discriminating BeII's stage II NEC from the benign form of pneumatosis intestinalis, NEC suspect, or spuriously suggestive GI conditions [86].

## 13. Liver Function Tests

Liver function tests (LFTs) incorporate albumin, alanine transaminase, aspartate transaminase, alkaline phosphatise, and total bilirubin. They allow physicians to gain information about the functional state of a patient's liver. Unsurprisingly, they often become deranged following the onset of NEC, but there is little evidence regarding their value in contributing to a diagnosis [87]. Unfortunately, many premature infants may already have abnormal LFTs because of parenteral feeding regimens; the link between PN and deranged liver enzymes is well established [88–91]. This could limit their usefulness in the diagnosis and monitoring of NEC.

## 14. The Use of Stool Inflammatory Markers inDiagnosing Necrotising Enterocolitis

Faecal calprotectin (FC) is a cytosolic component of neutrophils and is a useful marker for the exacerbation of inflammatory bowel disease. It is measured by a noninvasive biochemical test and is widely used to differentiate between functional bowel problems and inflammatory bowel disease. Thuijls et al. conducted a study in 2010 of 14 confirmed cases of NEC and concluded that faecal calprotectin is a potential diagnostic marker for NEC in neonates [92]. Its value in allowing early diagnosis of NEC has been alluded to by further trials [93, 94]. FC has been shown to be elevated in neonates with NEC but what remains unknown is its value in predicting the onset of NEC. Does the rise in FC precede clinical symptoms and other biochemical tests? Research involving larger cohorts is necessary to certify its true value and is an area of further interest to the author.

## 15. Umbilical Catheterisation 

There are conflicting reports of the extent to which umbilical catheterisation is potentially a risk factor for developing necrotising enterocolitis. Inherently it is extremely difficult to assess because of the challenges of isolating it as a variable. Early research showed an impairment in mesenteric blood flow was, associated with an umbilical catheter insertion [95]. *Roberton's Textbook of Neonatology* clearly states that it is a risk factor as do other sources [96]. However, other studies have shown the opposite to be true. Guthrie et al. studied 15072 neonates and found lower rates of NEC in those who had received an UAC at birth [97]. This conveys a potential protective effect of the catheters. However, a Cochrane review found that UAC was not a contributing factor in the aetiology of NEC [98]. With such conflicting research, the jury is clearly still “out” on the true risks of an umbilical catheterisation.

## 16. Clinical Risk Indicator in Babies

The Clinical Risk Index for Babies (CRIBs) score is a well-validated risk-adjustment instrument widely used in neonatal intensive care. Its appropriateness with contemporary data has been questioned so in 2003, a revised CRIB II score was developed [99]. This new scoring system was found to be a good predictive instrument for mortality in preterm infants by a large validation study in 2010 [100].

## 17. Congenital Disease (PDA)

The evidence behind a congenital persistent patent ductus arteriosis (PDA) as a cause for NEC is well established and has been confirmed by several prospective studies [101–103]. The left-to-right shunt that occurs in PDA results in the decreased velocity of mesenteric blood flow [104]. The intestinal mucosa has high metabolic activity and requires about 80% of total intestinal blood flow. When this is decreased, it becomes more susceptible to the disruption of its immune barrier functions [105].

## 18. Antibiotics

The immature immune system of preterm neonates puts them at the higher risk of neonatal sepsis. Benzylpenicillin and Gentamicin are given to most preterm babies because of the increased risk of sepsis. A recent retrospective cohort study involving 5693 premature babies found that the prolonged use (greater than 5 days) of antibiotic therapy was associated with and increased the risk of NEC [106]. The use of antibiotics for 5 days or less in premature infants was thoroughly assessed in a large RCT and no increase in the incidence of NEC was found between the control group and the study group [107].

## 19. Indomethacin

Exposure to indomethacin can occur prenatally as a tocolytic agent or postnatally to affect the closure of a PDA [108, 109]. The efficacy of this treatment has been demonstrated in several prospective trials [110, 111]. Perinatal exposure has been documented as a risk factor for intestinal injury in VLBW infants [112–114]. However, other studies have not been able to support these claims [115, 116]. With increasing usage, reports emerged the linking indomethacin use to isolated intestinal perforation (IIP) and necrotising enterocolitis [117–119]. However, most of these were retrospective studies and did not carefully differentiate between IIP and NEC. Furthermore, one of these studies failed to eliminate PDA as a confounding variable, a well-documented risk factor for NEC [120]. In contrast, a thorough prospective analysis over 12 years into the exact effect of perinatal indomethacin use found a positive association with IIP but a negative association with NEC using multivariate logistic regression analysis (exposed: 14.6% unexposed: 9.9%) [121]. These findings were independent of maternal milk feeding and have been supported by subsequent research [122]. It appears therefore that the protective benefits of indomethacin for NEC have potentially been masked by confounders such as IIP and PDA.

## 20. Dexamethasone

Chronic lung disease (CLD) remains a major problem in neonatal intensive care units. The most likely underlying pathogenesis involves persistent inflammation in the lungs. Corticosteroids have been used to either prevent or treat CLD because of their potent anti-inflammatory effects [123]. Early studies appeared to show a link between dexamethasone use and NEC, one reporting a 7.4% increase in the group exposed to steroids [124]. Increased frequency of sepsis and neonatal infections was also seen in this group. Despite this finding, many other studies have reported an overall decrease in perinatal mortality in the group treated with steroids [125]. More recently, the topic of steroid use perinatally has been the focus of an extensive Cochrane review. It demonstrated that steroids are effective both prenatally and/or postnatally in promoting lung maturation, and that they were not found to be associated with increased incidence of NEC [126, 127].

Other identified risk factors that are not possible to discuss within the confines of this report include maternal cocaine use, variations in blood glucose levels, sepsis, IgA supplementation, and the stool cultures of pathogenic micro-organisms [128, 129].

## 21. Conclusions

This paper highlights just one of the many challenges involved in neonatal intensive care. Necrotising enterocolitis has proven itself as an immensely enigmatic and morbid disease, the aetiology of which is tied up in a minefield of medical literature, much of it conflicting in its findings. This paper has demonstrated the paramount importance nutrition in the first few weeks of life and what a fine balance needs to be struck between malnourishment and high risk aggressive feeding. 

There still remain many potential risk factors for NEC; however, the extent to which these risk factors are important in the aetiology of NEC continues to elude medical research. It seems that no individual factor alone is sufficient to precipitate NEC pointing to a multifactorial aetiology. However, with the management of premature neonates being aggressively monitored and standardised, possibilities or a more intrinsic nutrient-gene interaction arises, which we have yet to understand. By understanding this in the future, we might be able to develop targeted therapies for individuals who are most susceptible to NEC.

## Figures and Tables

**Table 1:** From *Roberton's Textbook of Neonatology*. Risk factors for necrotising enterocolitis.

Prematurity
Intrauterine growth restriction
Placental abruption
Premature rupture of membranes
Perinatal asphyxia
Low Apgar score
Umbilical catheterisation
Hypoxia and shock
Patent ductus arteriosus
Hypertonic feeds
Nonhuman milk formula
Rapid introduction of enteral feeds
Fluid overload
Pathogenic bacteria
Polycythaemia
Thrombocytosis
Anaemia
Exchange transfusion
Cyanotic congenital heart disease

## Abbreviations

NEC: Necrotising enterocolitis
EBM: Expressed breast milk
DEBM: Donor expressed breast milk
RACH: Royal Alexandra children's hospital
RSCH: Royal Sussex County hospital
TPN: Total parenteral nutrition
EN: Enteral nutrition
RDS: Respiratory distress syndrome
CLD: Chronic lung disease
PDA: Patent ductus arteriosus
IVH: Intraventricular haemorrhage
BPD: Bronchopulmonary dysplasia
GOR: Gastro-oesophageal reflux
PTX: Pneumothorax
FC: Faecal calprotectin
IIP: Isolated intestinal perforation.
